# Effect of strength training on sleep apnea severity in the elderly: study protocol for a randomized controlled trial

**DOI:** 10.1186/s13063-017-2238-3

**Published:** 2017-10-23

**Authors:** Roberto Pacheco da Silva, Denis Martinez, Pedro Lopez, Eduardo Lusa Cadore

**Affiliations:** 1 Graduate Program in Cardiology and Cardiovascular Sciences, Universidade Federal do Rio Grande do Sul (UFRGS), Porto Alegre, RS Brazil; 2 Cardiology Unit, Hospital de Clinicas de Porto Alegre (HCPA), UFRGS, Porto Alegre, RS Brazil; 3 Exercise Research Laboratory, UFRGS, Porto Alegre, RS Brazil; 4 Physical Education School, UFRGS, Porto Alegre, RS Brazil

**Keywords:** Exercise, Training, Sleep apnea, Elderly, Strength, Functional capacity

## Abstract

**Background:**

Obstructive sleep apnea (OSA) occurs due to sleep-induced upper airway muscle relaxation resulting in increased pharyngeal collapsibility. Clinical trials have shown a favorable effect of exercise training on OSA severity in middle-aged adults. Aging is characterized by motor-unit loss. Force training may affect the whole body muscle tone. We hypothesize that interventions increasing muscle strength might propagate to motor units at the abductor pharyngeal muscles, reducing collapsibility and, hence, sleep apnea severity in elderly patients with obstructive sleep apnea.

**Methods/design:**

This is a randomized clinical trial including patients between 65 and 80 years of age, with obstructive sleep apnea, and an apnea-hypopnea index (AHI) between 20 and 50 events/hour, diagnosed by out-of-center in-home type III polysomnography. Forty subjects will be included and randomly assigned to two equal sized groups. The participants allocated to the intervention group will attend two sessions per week of one-hour strength training for the legs, arms, chest, back, and abdomen and the controls will receive advice on lifestyle change. The primary outcome measure of the study will be the change in apnea-hypopnea index and the secondary outcomes will be the body composition, evaluated by anthropometric and bioelectrical impedance variables; maximum dynamic force, appraised by one-repetition maximum strength test; muscle quality and thickness by ultrasound; physical function assessed by sit-to-stand test, timed up and go test, handgrip strength test. The study duration will be 12 weeks. Intention-to-treat and per-protocol analyses will be performed.

**Discussion:**

The high prevalence of obstructive sleep apnea in elderly people is a public health issue. OSA is a recognized cause of cardiovascular disease and reduces quality of life due to sleepiness and fatigue. Exercise is a low-cost intervention that could help to detain the trend towards age-dependent loss of pharyngeal motor units and progressive severity of obstructive sleep apnea. Home-based strength exercises may represent a more practical approach than aerobic exercise for elderly patients. If the results confirm our hypothesis, further research on the clinical application of our findings will be warranted.

**Trial registration:**

ClinicalTrials.gov, NCT02742792. Registered on 1 April 2016.

**Electronic supplementary material:**

The online version of this article (doi:10.1186/s13063-017-2238-3) contains supplementary material, which is available to authorized users.

## Background

Obstructive sleep apnea (OSA) causes repeated surges of hypoxia and awakenings during sleep [1]. OSA occurs in up to one third of the population [2], constituting a public health problem [3]. The prevalence of sleep apnea increases with age. In young women, the prevalence is 1.4%. In both men and women 70 years or older, it reaches 90% and more [2, 4]. This remarkable increase in prevalence is explainable, at least in part, by escalating body weight with age, but several contributing factors, which deserve medical attention, should be considered.

The repeated arousals caused by the need to restore breathing after each one of the recurrent breathing interruptions have as a consequence sympathetic hyperactivity, somnolence, and fatigue. Intermittent hypoxia is associated to oxidative stress, inflammation, and cardiovascular impairments [5]. Periods of sleep-dependent hypoxia also cause motor cortex dysfunction [6] Intermittent hypoxia was shown to cause demyelination [7] and neuronal damage, notably in the cerebellum [8]. In addition, OSA is a risk factor for hypertension [9], coronary artery disease [10] , heart failure [11], and arrhythmias [12].

Treatment options for OSA are several and the most effective is the use of a continuous positive airway pressure (CPAP) device [13]. Oral appliances for mandibular advancement [14, 15], surgery [16], weight reduction, and lifestyle change, including recommendation of regular exercise [17] are alternatives to CPAP.

Physical exercise is accepted culturally and scientifically as a non-pharmacological intervention beneficial to health [18, 19]. Aerobic and resistance training improves general wellbeing and particularly sleep quality [20, 21]. A sedentary lifestyle is linked with higher prevalence and severity of sleep apnea. On the other hand, exercise gives consistent results in treating OSA, and increasing the number of hours of exercise is associated with reduction in the severity of OSA [22, 23].

In experimental studies, the effect size of exercise in the treatment of OSA ranges between 0.6 [24] and 1.5 [25] standard deviations. In a meta-analysis of five studies, totaling 129 participants, the mean effect is a reduction of seven events per hour [26]. All randomized clinical trials and other studies have involved participants between 42 and 54 years of age on average. No study involving the most affected population, elderly individuals, was identified in extensive PubMed and Embase searches. The decrease in physical fitness in the elderly is associated with motor unit loss [27, 28], falls [29, 30], pain [31], musculoskeletal changes, and sleep disorders [32, 33]. Moreover, the worsening of overall physical function is a predictor of mortality [34].

The relevance of strength training in elderly people is linked to the medical consequences of muscle fiber loss. Sarcopenia starts at the age of 35 years and 45 years, in men and women, respectively [35–37], and progresses at a rate of 1–2% per year after the sixth decade [38]. It is aggravated by sedentary lifestyle, reduced levels of trophic hormones, and neurodegenerative processes [39, 40]. It is plausible that weakening of the pharyngeal abductor muscles participates in the age-related increase in the prevalence of OSA. Hypothetically, a widespread tonic effect [41] could lead to improvement in pharyngeal abductor function, bettering airway patency during sleep.

The increase in OSA prevalence with ageing may be linked also to overnight rostral fluid displacement from the legs [42]. Leg fluid volume correlates with the apnea-hypopnea index (AHI) and with the number of hours sitting, suggesting that “a sedentary way of life may predispose to OSA” [43].

Despite the abundant evidence on the effects of resistance training on the neuromuscular function, the issue of deleterious effects of OSA on muscle function in elderly subjects remains uninvestigated. Moreover, no high-level evidence on the effects of strength training on the severity of OSA in elderly people was found.

### Rationale

The systemic improvement in muscle strength after resistance training may reach the abductor pharyngeal muscle and improve OSA. Hypothetically, a widespread tonic effect would lead to an improvement in pharyngeal abductor function and bettering of airway patency during sleep. Also, reduction in sedentarism may reduce fluid retention. Reduction in the severity of OSA induced by physical training has been shown in randomized clinical trials and meta-analyses, but none of the studies to date have investigated the effect of strength training on sleep apnea severity in elderly individuals.

### Research question

Will elderly people performing resistance training reduce the AHI in comparison with a control group performing recreational physical activity?

## Methods/design

### Study design

This study is a parallel randomized, blind, controlled superiority trial. The study strategy is registered, constructed and presented according to the recommendations for interventional trials [44] (see Additional file 1 for the Standard Protocol Items: Recommendations for Interventional Trials (SPIRIT) checklist).

#### Eligible participants

##### Inclusion criteria

Patients should fulfill the following criteria:

- Age between 65 and 80 years
- Either gender
- Not being engaged in regular structured resistance exercise
- AHI between 20 and 50 events per hour
- Availability of time to include physical activity in their routine
- Consenting to participate in the survey

##### Exclusion criteria

- Being in treatment for sleep apnea
- Osteoarticular injuries or illnesses that impair the performance of the exercises included in the project
- Neuromuscular problems
- Uncontrolled hypertension
- Acute myocardial infarction or stroke in the last year
- Recent trauma to the upper airway
- Other serious chronic disease with treatment for over a month in the last year
- Regular or abusive use of medications or drugs with effects on the central nervous system

### Sample size

The sample size calculation for this trial was performed using the G-Power program (Franz Faul, Universität Kiel, Germany) [45]. We chose an effect size of 0.6 standard deviations described as the lowest among five articles included in a meta-analysis of the effect of exercise studies on sleep apnea [24] as the target for our study. For two groups (training and control) and two assessments (before and after 12 weeks of training), with 90% power and probability of alpha error of 5%, the sample size calculated for analysis of variance with repeated measures would be 16 patients in each group. To compensate for possible participant loss, 40 subjects will be included. Losses after randomization will be included in the intention-to-treat but not in the per-protocol analyses.

### Randomization process

Randomization will be performed using a sequence of numbers generated by computer at randomization.com. Patients will be assigned to the intervention or control groups by a researcher with no information about the participants and not otherwise involved in the protocol.

### Blinding

The evaluators taking measurements for outcomes will be blinded to the group assignment. The physical educator responsible for prescribing and monitoring the training will not be blinded to the groups and will not be involved in other steps of the protocol. A certified scorer blinded to the groups will perform the scoring of the AHI. The blinding code will be broken at the end of the study or earlier by request of regulatory committees or in the case of serious adverse events in connection with the group assignment.

### Recruitment

The information technology system of the university hospital will generate a list of patients aged 65 to 80 years ascribed to the institutional primary care unit. After chart review, the subjects will be invited to join in the study through telephone calls. Those willing to participate will visit the institutional research center to undergo the informed consent process and initiate the assessment.

### Statistical methods

Intention-to-treat and per-protocol analyses will be performed for the primary and secondary outcomes. Patients who do not perform the 24 training sessions or who report poor adherence to the healthy lifestyle program during 2 weeks or more will be excluded from the per-protocol analysis. Missing data will be considered as missing, without data imputation.

Means and standard deviation will be used to represent data with a normal distribution and medians and quartiles will be used to describe non-normally distributed data. Natural logarithm transformation will be used before inclusion of non-normally distributed dependent variables in analyses. The significance of the differences between groups, at the baseline, will be tested by the Student *t* test and chi-squared test, for linear and categorical variables, respectively. Generalized estimating equations (GEE) will be used to detect differences between exercise and control groups and time*group interaction. The results with *P* values <0.05 for alpha error will be considered statistically significant. Statistical analyses will be performed using SPSS software (SPSS Inc., Chicago, IL, USA).

### Monitoring

Monitoring of the physical activity in the intervention group will be performed by the physical educator after each session in a specific form of the electronic case report form. Patients in the control group will record in a log sheet the number of goals met during each week. The institutional ethics committee will be monitoring the data. All analyses will be performed at the end of the study.

### Study measurements

#### Description of intervention, comparison group, and follow up

**Fig. 1:**
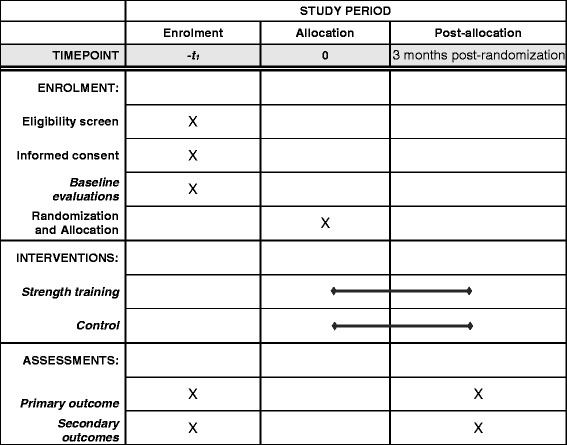
Standard Protocol Items: Recommendations for Interventional Trials (SPIRIT) figure

**Fig. 2:**
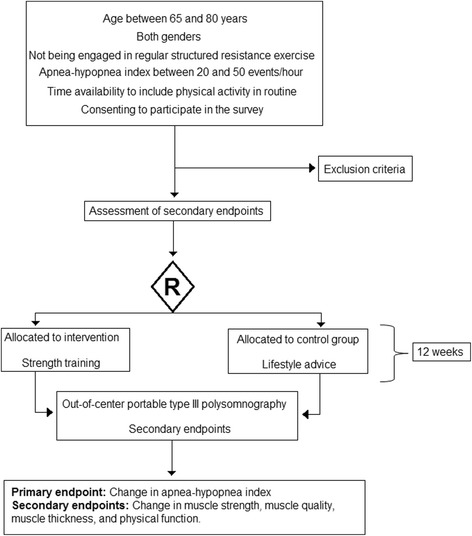
Flow diagram of the study design

The patients will be allocated to strength training or lifestyle advice groups. A detailed description of the phases of the study are shown in Figs. 1 and 2.

The program will consist of a twice-a-week routine of strength training sessions over 12 weeks. The sessions will be separated by at least 48-hour intervals. Each training session will consist of exercises for the legs, arms, chest, back, and abdomen. The subjects will be instructed to perform the concentric and eccentric phases of each repetition with controlled speed (2–3 seconds at each stage). The training will be divided into cycles of 4 weeks each with intensity and progressive exercise volumes.

- First cycle: two sets of 12–14 repetitions in the first week using 50% maximal load in order to improve the execution and motor control of exercises, followed by 3 weeks in which individuals will perform three sets of 12–14 repetitions maximum (i.e., performance of sets until concentric failure).
- Second cycle: three sets of 10–12 repetitions maximum.
- Third cycle: three sets of 8–10 repetitions maximum.

The rest interval between sets will be 1 to 1.5 minute [46, 47]. The load for each exercise will be adjusted according to the maximal performance of each individual.

The individuals allocated to the control group will receive advice on lifestyle. In addition, individuals will be encouraged to participate in the meetings of an elderly-directed activity group in the primary care unit of the university hospital.

### Ethics

When the subject enters the study, the procedures performed in the physical tests, bio impedance, polysomnography, and training will be explained in detail. The anticipated risks to participants, beyond the occasional pain due to the training effort, will be fatigue and/or dizziness during and/or at the end of exercises. All these signs and symptoms are expected and reversed after cessation of effort. Post-delayed muscle pain, lasting up to 48 hours after the exercise session is a normal effect of strength training, mainly after the first sessions of training. The patient can leave the trial at any time.

The subjects identified with AHI <20 or AHI >50, who are excluded from the study, will be submitted for consultation and treatment in the sleep research clinic in the primary care unit. At the end of the study, all volunteers will be forwarded to the sleep research clinic in the primary care unit for medical treatment.

The trial results will be published in an international journal with impact. The investigators will communicate the trial results to participants via email. No publication restrictions will be implemented.

### Outcomes

#### Primary outcomes

- Apnea-hypopnea index (number of apnea and hypopnea events per hour of artifact-free recording in the polygraphy)

#### Secondary outcomes

- Muscle strength
- Muscle thickness
- Muscle quality
- Body composition (fluid, fat)
- Physical function

### Follow up and duration of the study

There will be four visits at the clinical research center for outcome evaluation. Two will be at baseline (a visit to receive the polygraph device and another visit for carrying out evaluation of body composition, physical function, and muscle strength, function, and quality) and two visits at the final follow up (reassessment of the same parameters). The study protocol consists of 12 weeks of strength training twice a week (24 sessions).

### Assessment of outcomes

#### Apnea-hypopnea index

Out-of-center polygraphy will assess the presence and severity of sleep apnea. Portable type III polysomnography monitors (Embletta Gold III, Embla, Broomfield, CO, USA; or SomnoCheck Effort, Weinmann, Hamburg, Germany) will be employed to evaluate: (1) chest effort, (2) abdominal effort, (3) oximetry, (4) nasal airflow, and (5) position, as previously described [48]. Apnea and hypopnea events, respiratory effort related arousals (RERAs), and the apnea-hypopnea and respiratory disturbance index will be reported according to the rules of the American Academy of Sleep Medicine (AASM) [49]. The AHI will define the diagnosis of sleep apnea. Polysomnography will be performed before and after 12 weeks using the same polygraph monitor on both occasions.

### Muscle strength

Subjects will perform one-repetition maximum (1-RM) tests of knee extension (unilateral) and preacher curl elbow flexion (bilateral) (Können Gym, Porto Alegre, Brazil). The same investigator, with identical subject/equipment positioning, will conduct the pre-tests and post-tests. Before the 1-RM tests, subjects will be familiarized with the testing procedures and will perform 10 repetitions with light resistance as warm up. Thereafter, resistance will be increased until the subjects became unable to lift the additional weight using the proper technique. The time spent in each muscle action will be controlled (~2 seconds in both concentric and eccentric phases). All 1-RM values will be determined within 3–5 attempts, with 3 min rest between each attempt. At post-testing, 1-RM will be performed 3–5 days after the last training session.

### Muscle thickness

Quadriceps femoris B-mode ultrasound images will be obtained with a 38-mm, 9.0 MHz linear-array probe (image 70 mm depth; 90 dB general gain, time gain compensation (TGC) at a neutral position) with a Nemio XG ultrasound device (Toshiba, Japan). Before any measurement, subjects will rest in the supine position with the lower limbs extended and relaxed for 15 min to allow fluid shifts to stabilize [50]. Transversal images of the right vastus lateralis (VL), rectus femoris (RF), vastus intermedius (VI) and vastus medialis (VM) muscles will be acquired. The probe will be coated with a water-soluble transmission gel to provide acoustic contact and care will be taken to avoid the compression of the dermal surface. The measurement sites will be the same as those adopted in previous studies [51–54]. All images will be acquired and analyzed by the same trained investigator. At follow up, 3–5 days after the last training session, muscle thickness will be measured.

The computer-assisted determination of muscle thickness will use the standard function of ImageJ 1.42q software (National Institute of Health, USA). The calculation will include the distance between adipose tissue-muscle interface for VL, RF, and VM, and bone-muscle interface for VI. Whole quadriceps muscle thickness will be obtained as the sum of the four individual quadriceps portions:

$$ \mathrm{QMT}=\left(\mathrm{VL}\ \mathrm{muscle}\  \mathrm{thickness}+\mathrm{RF}\ \mathrm{muscle}\  \mathrm{thickness}+\mathrm{VI}\ \mathrm{muscle}\  \mathrm{thickness}+\mathrm{VM}\ \mathrm{muscle}\  \mathrm{thickness}\right) $$

### Muscle quality

#### Echo intensity

Echo intensity (EI) will be determined according to previous studies [52, 53] by computer-assisted grayscale analyses using the standard function of ImageJ 1.42q software (National Institute of Health, USA). Single images of each muscle will be digitized and analyzed. Regions of interest of each quadriceps muscle portion (VL, RF, VI, and VM) will be selected, including as much muscle as possible but avoiding other tissues (such as bone and surrounding fascia) for EI calculation of each component of the quadriceps femoris. The mean EI will be determined using a standard gray-scale histogram function and expressed as a value between 0 (black) and 255 (white). At follow up, EI will be performed 3–5 days after the last training session. Thereafter, the EI will be determined from the average EI value from all quadriceps muscle portions:

$$ \mathrm{EI}=\left(\mathrm{VL}\ \mathrm{echo}\  \mathrm{intensity}+\mathrm{RF}\ \mathrm{echo}\  \mathrm{intensity}+\mathrm{VI}\ \mathrm{echo}\  \mathrm{intensity}+\mathrm{VM}\ \mathrm{echo}\  \mathrm{intensity}\right)/4 $$

#### Specific tension

To obtain a value for specific tension (ST), the knee extension 1-RM value will be divided by the muscle mass unit. Thus, the ST will be determining according to previous studies [53] using o the following equation:

$$ \mathrm{Specific}\  \mathrm{Tension}=1\hbox{-} \mathrm{RM}\ \left(\mathrm{kg}\right)/\mathrm{QMT}\ \left(\mathrm{mm}\right) $$

### Physical function

Physical function will be evaluated by a battery of three tests: (1) handgrip strength, (2) sit-to-stand ability, and (3) mobility.

#### Maximal handgrip strength

The maximal handgrip strength will be determined in the dominant arm using a handgrip dynamometer (Jamar Hydraulic Hand Dynamometer, Sammons Preston CO, Bolingbrook, IL, USA) [55]. The maximal strength will be measured three times at intervals of 2 min of rest between assessments. The maximal value obtained will be used as maximal strength [56].

#### Sit-to-stand ability

Sit-to-stand ability will be assessed by the sit-to-stand test [57, 58]. The test includes five complete movements of getting up and sitting in the chair during the shortest possible time [59, 60].

#### Mobility

To evaluate mobility, the timed up and go test will be used [61–63]. The performance and time during the test reflects reaction time, muscle strength of the lower limbs, balance disorders and difficulty walking [64, 65]. This test evaluates the execution speed in getting up from a chair with arms, walking ahead 3 m, turning around, walking back, and sitting in the chair [66].

## Discussion

The present study is designed to evaluate the effects of resistance training on OSA severity in elderly persons. Our hypothesis is that resistance training will reduce OSA severity while improving neuromuscular function. A previous systematic review and meta-analysis showed that exercise training reduces approximately seven events in AHI compared to control groups [24]. However, little is known about the effect of resistance training alone without aerobic training on OSA in the elderly population. Also, the efficacy of this intervention remains untested in elderly populations, possibly because of prejudices about age-related frailty. None of the studies found in an ample PubMed and Embase search mentioned any randomized controlled trials (RCTs) of treatments for OSA

Resistance training is recognized as the most effective intervention to improve the muscle mass and quality, i.e., lean muscle mass, quadriceps muscle thickness, and intramuscular adipose tissue. Also, resistance training enhances the neuromuscular function, i.e., lower limb strength, and specific tension in elderly persons [67]. To the best of our knowledge this would be the first study to investigate the heterotopic effect of resistance training. Regarding the secondary outcomes, the enhancements of performance in functional tests have seldom been described in elderly people [68]. Therefore, our RCT will provide data for analyses of the relationship between OSA and functional performance.

### Trial status

At the time of manuscript submission, the enrollment of volunteers is ongoing.

## Additional file

Additional file 1: SPIRIT checklist. (DOC 121 kb)

## Abbreviations

AHI: Apnea-hypopnea index
CPAP: Continuous positive airway pressure
EI: Echo intensity
OSA: Obstructive sleep apnea
RCT: Randomized controlled trial
RF: Rectus femoris
SPIRIT: Standard Protocol Items: Recommendation for Interventional Trials
ST: Specific tension
VI: Vastus intermedius
VL: Vastus lateralis
VM: Vastus medialis
